# The role of biophysical cohesion on subaqueous bed form size

**DOI:** 10.1002/2016GL067667

**Published:** 2016-02-19

**Authors:** Daniel R. Parsons, Robert J. Schindler, Julie A. Hope, Jonathan Malarkey, Jaco H. Baas, Jeffrey Peakall, Andrew J. Manning, Leiping Ye, Steve Simmons, David M. Paterson, Rebecca J. Aspden, Sarah J. Bass, Alan G. Davies, Ian D. Lichtman, Peter D. Thorne

**Affiliations:** ^1^ Department of Geography, Environment and Earth SciencesUniversity of HullHullUK; ^2^ School of Marine Science and EngineeringPlymouth UniversityPlymouthUK; ^3^ School of BiologyUniversity of St AndrewsSaint AndrewsUK; ^4^ School of Ocean SciencesBangor UniversityAngleseyUK; ^5^ School of Earth and EnvironmentUniversity of LeedsLeedsUK; ^6^ HR WallingfordWallingfordUK; ^7^ Centre for Applied Marine SciencesBangor UniversityAngleseyUK; ^8^ National Oceanography CentreLiverpoolUK

**Keywords:** sediment, bed forms, cohesivity, roughness, biophysical

## Abstract

Biologically active, fine‐grained sediment forms abundant sedimentary deposits on Earth's surface, and mixed mud‐sand dominates many coasts, deltas, and estuaries. Our predictions of sediment transport and bed roughness in these environments presently rely on empirically based bed form predictors that are based exclusively on biologically inactive cohesionless silt, sand, and gravel. This approach underpins many paleoenvironmental reconstructions of sedimentary successions, which rely on analysis of cross‐stratification and bounding surfaces produced by migrating bed forms. Here we present controlled laboratory experiments that identify and quantify the influence of physical and biological cohesion on equilibrium bed form morphology. The results show the profound influence of biological cohesion on bed form size and identify how cohesive bonding mechanisms in different sediment mixtures govern the relationships. The findings highlight that existing bed form predictors require reformulation for combined biophysical cohesive effects in order to improve morphodynamic model predictions and to enhance the interpretations of these environments in the geological record.

## Key Points

- Subaqueous bed form dimensions are controlled more by biological than physical cohesion
- Existing predictors require reformulation for combined biophysical cohesive effects
- Small amounts of substrate biological cohesion act to reduce bed roughness 2 orders of magnitude

## 1 Introduction

Estuaries, the coastal zone, and the continental shelf make up 8% of the world's oceans [*Meadows et al.*, 2012], and in these environments subaqueous dune bed forms are the primary sedimentary structures, acting as principal contributors to bed roughness and mediating sediment fluxes [e.g., *Lefebvre et al.*, 2011; *Naqshband et al.*, 2014]. These coastal and nearshore locations are among the most sensitive regions in terms of sea level rise, a problem exacerbated by the predicted increased frequency of extreme weather events, which will act to alter a range of sediment transport processes [e.g., *FitzGerald et al.*, 2008]. Consequently, a robust understanding of how dune dimensions relate to controlling hydrodynamics is crucial for informing estuarine and coastal management, including prediction of the impacts of sea level rise, the maintenance of navigable channels [*van der Mark et al.*, 2008], scour around engineering infrastructure, and roughness parameterizations in numerical models [*Ganju and Sherwood*, 2010]. Understanding the transport of organic material is important for determining ecological interactions and overall organic carbon fluxes [*Battin et al.*, 2008] in the coastal zone, and habitat modeling also requires appropriate dynamic models of bed form development in order to better predict spatial distributions of biological activity [e.g., *Habersack et al.*, 2014]. Finally, preserved bed forms in the geological record are first‐order predictors for minimum water depth and hence environmental reconstruction [e.g., *Leclair and Bridge*, 2001].

Prediction of sediment transport rates presently relies on bed form phase diagrams and empirical bed form prediction formulae that are based exclusively on cohesionless silt, sand, and gravel [e.g., *van den Berg and van Gelder*, 1993; *van Rijn*, 1984]. However, substrates composed of mixtures of sand and mud are common to many coasts, deltas, estuaries, and lowland rivers [*Healy et al.*, 2002]. The organic portion of these habitats is often ignored in sedimentological studies, but the importance of living organisms, their products, constructions, and remains can strongly mediate the physical behaviors and functionality of depositional systems [*Black et al.*, 2002]. Substrata composed of sand‐mud mixtures are important habitats for benthic biota. Moreover, where light penetrates to the bed, microbial communities driven by oxygenic photosynthesis [*Staats et al.*, 2000] also have the capacity to alter the surrounding physical and chemical nature of the substratum [e.g., *Meadows et al.*, 2012]. Many subaqueous environments consist of mixtures of cohesionless sand, physically cohesive mud, and benthic organisms. The latter can increase sedimentary stabilization via burrow formation, cast constructions, and more pervasively, the secretion of cohesive extracellular polymeric substances (EPS) [*Tolhurst et al.*, 2002]. As a result, the erosion thresholds of both cohesive and noncohesive sedimentary fractions are known to significantly increase in the presence of EPS [e.g., *Tolhurst et al.*, 2002]. Most studies have focused on the mechanical protection derived from high concentrations of EPS in the form of surface biofilms, where surface scour is more likely than bed form development [*Hagadorn and McDowell*, 2012]. However, EPS are also distributed at lower concentrations (0.01–0.1%) throughout the sediment substratum [*Lanuru et al.*, 2007], where their influence on bed form development has been observed experimentally [*Malarkey et al.*, 2015].

A handful of studies has examined sediment transport and bed forms in cohesive sediment under controlled conditions. However, these studies have treated biological and physical cohesion separately [*Baas et al.*, 2013; *Malarkey et al.*, 2015; *Schindler et al.*, 2015]. While these studies have elucidated the differences between biological and physical cohesion, their applicability to the natural aquatic environment is limited, because physical and biological cohesion almost always occur together [*Friend et al.*, 2008]. Furthermore, the interaction between physical and biological cohesion for bed form dynamics is largely unknown. Here we provide the first results under controlled conditions of the influence of physical and biological cohesion on equilibrium dune morphology by means of laboratory experiments and examine the nature of the cohesive bonding mechanisms in three‐way mixtures of mud, sand, and EPS.

## 2 Methods

Experiments were undertaken in a recirculating flume channel, 10 m long and 2 m wide, in the Total Environment Simulator at the University of Hull. Uniform flow conditions were maintained over the test section (Figure S1 in the supporting information), and the flow velocity was monitored at an acquisition rate of 25 Hz throughout each experimental run, using four vertically stacked 10 MHz acoustic Doppler velocimeters (ADVs) located close to the flume centerline (Figure S1). Flow depth (*d*) was 0.38 m for all runs, and depth‐mean flow velocity (*U*) over an initially flat bed was 0.80 m s^−1^, yielding subcritical and fully turbulent flow. Salinity was set, using sodium chloride, to approximate estuarine conditions, at 16 practical salinity units (psu), which is equivalent to a density of 1010 kg m^−3^. These experimental settings, together with the mean grain size of the substrata used, are known to generate three‐dimensional equilibrium dunes [*van den Berg and van Gelder*, 1993] (Figure S2).

Three types of substrata were prepared, corresponding to series A–C (Table 1). In series A, only physical cohesion was considered [*Schindler et al.*, 2015], with sand‐mud mixtures made using two sediment fractions: upper fine sand with a median diameter, *D*_50_, of 239 µm and kaolinite clay with a *D*_50_ of 3.4 µm. Seven substrata, labeled runs A1–A7, were prepared by incrementally increasing initial substratum mud content (1.9% < *m* < 14.1% by dry weight). In series B and C, various ratios of sand, mud, and EPS were combined to form a homogenous mixture. Xanthan gum was used as a proxy for EPS found in natural sediment [e.g., *Tolhurst et al.*, 2002]. The range of EPS content used in the experiments is comparable with background ranges measured at intertidal sites in the Eden and Dee Estuaries, U.K. (0–0.1% EPS per dry weight of sediment), collected as part of parallel field investigations [*Malarkey et al.*, 2015], which tended to be relatively constant with depth in the upper centimeter of the bed. The initial and final depth‐mean EPS contents were determined by applying the phenol‐sulphuric acid assay [*Dubois et al.*, 1956] to millimetric slices taken from 100 mm long, 10 mm diameter syringe core samples. Standard grain size analysis techniques were used to quantify bed mud contents after each experiment.

**Table 1:** Experimental Parameters for Series A–C^a^

Run	*m* (%)	*e* (%)	*H* (mm)	*L* (mm)	*H*/*L* (−)	*k_s_* (mm)
A1	1.9	0.0	75	1549	0.0482	90.77
A2	4.7	0.0	65	1135	0.0571	94.07
A3	8.9	0.0	25	1011	0.0252	17.87
A4	9.8	0.0	24	894	0.0266	16.18
A5	11.9	0.0	22	741	0.0292	16.41
A6	12.7	0.0	11	625	0.0170	4.91
A7	14.1	0.0	18	537	0.0326	14.37
B1	2.8	0.027	37	990	0.0372	39.85
B2	6.8	0.038	13	772	0.0170	5.58
B3	15.4	0.030	4	979	0.0042	0.44
C1	9.1	0.075	4	121	0.0364	3.99
C2	9.9	0.071	4	116	0.0332	3.19
C3	12	0.073	3	115	0.0275	2.16
C4	17.7	0.100	‐	‐	‐	‐

a *m* is initial bed mud fraction, *e* is initial bed EPS fraction, *H* is mean bed form height, *L* is mean bed form length, *H*/*L* is bed form steepness, and *k_s_* = 25*H* ^2^/*L* is bed roughness.

In series B, EPS was added at a low concentration (mean EPS content *e* = 0.032 ± 0.006%) in order to represent environments with low primary production rates [*Lanuru et al.*, 2007]. Three substrata with increasing mud content were made (2.8 < *m* < 15.4%). In series C, EPS content was set to approximately 3 times the concentrations in series B (mean *e* = 0.086 ± 0.015%) to represent environments with high primary production rates [*Lanuru et al.*, 2007]. Four substrata with increasing mud content were made (9.1 < *m* < 17.7%). Each substratum was manually flattened across the whole flume to a thickness of 0.20 m and subjected to the 0.80 m s^−1^ flow for a period of 10.5 h.

Bed topography was measured across a swathe of the channel bed at the end of each experiment, using a 2 MHz ultrasonic ranging sensor system mounted on an automated traverse oriented along the center of the flume, spanning a test section distance of 4.7 m (Figure S1). The dune dimensions were quantified from three transects in the swathe. Bed form length (*L*) was defined as the distance between consecutive crests, and height (*H*) was defined as the vertical distance from the crest to the upstream trough. Some dunes exhibited superimposed ripples. Dunes were distinguished from ripples by their order of magnitude longer lengths and laterally continuous crest lines that stretched across the width of the channel [cf. *Reesink and Bridge*, 2007]. The height and length of each bed form along each transect were averaged together to produce a representative height, *H*, and length, *L*, for each experiment.

In order to examine the nature of the cohesive bonding mechanisms, bed samples were also taken prior to the experiments in all three series of runs and compared using low‐temperature scanning electron microscopy (LTSEM) [*Paterson*, 1995].

## 3 Results

**Figure 1:**
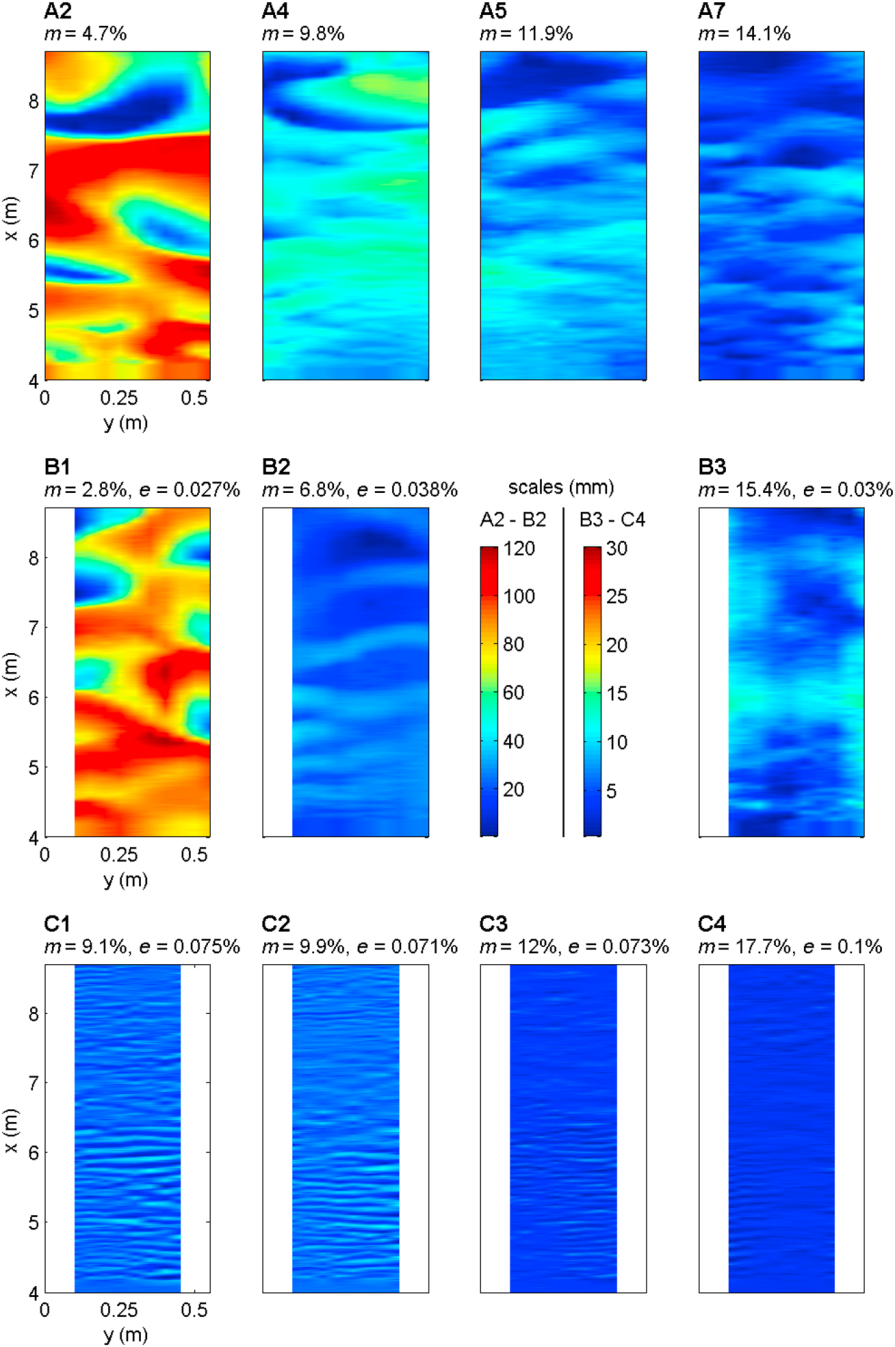
Planform contour maps of the final bed morphology of the experimental runs taken over a central swath of the test domain (*x* is the distance downstream). First row shows selected runs for series A (no EPS), where a reduction in bed form dimensions occurs as mud content is increased, resulting in a transition from fully three‐dimensional dune‐scale bed forms via ripples superimposed on dunes to surfaces that approach a flat bed. The second row shows bed forms from series B (low EPS). These bed forms are small compared with series A, and a transition from irregular, low‐steepness 3‐D dunes (run B1) to an almost featureless surface (B3) is evident. The third row show bed forms from series C (high EPS). These bed forms are limited to 2‐D ripples and approach a featureless surface at the highest mud content (run C4). *m* = initial bed mud content; *e* = initial bed EPS content. Note the dramatic changes in bed form type and size for mere trace amounts of EPS.

**Figure 2:**
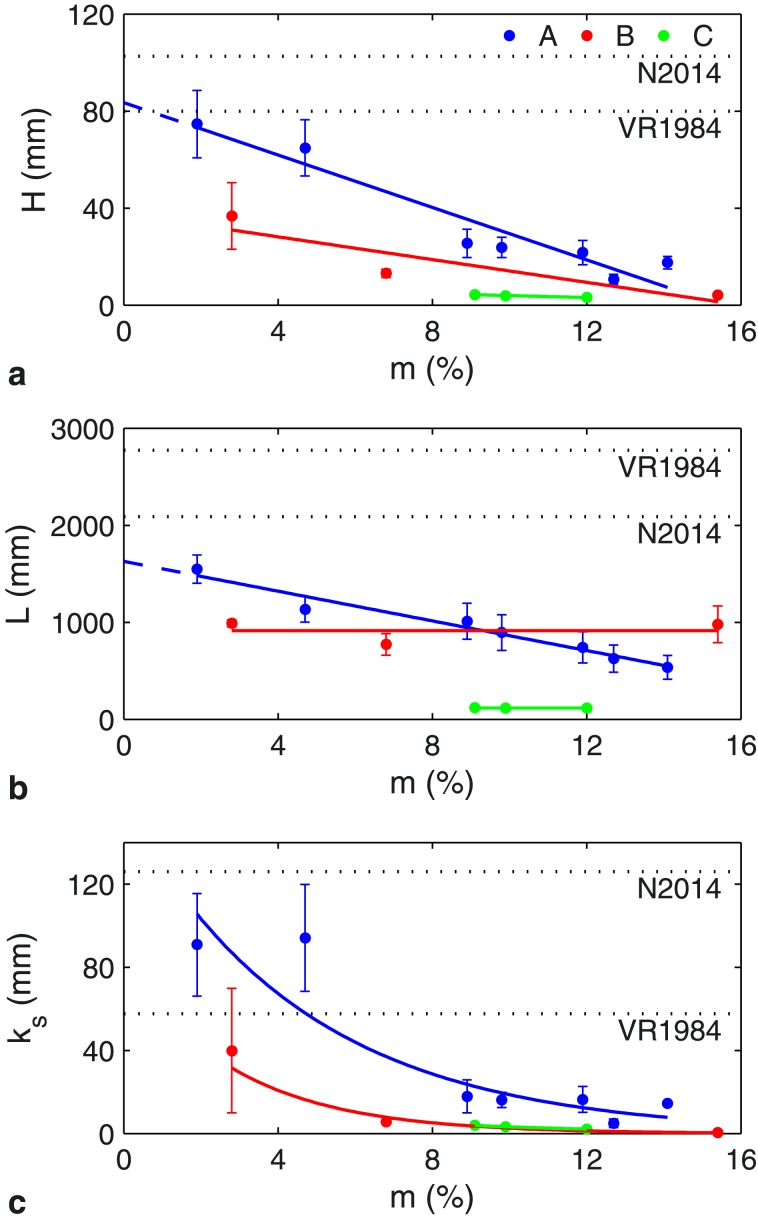
(a) Relationship between bed form height, *H*, and initial mud content, *m*, for series A (blue, no EPS), B (red, low EPS), and C (green, high EPS). (b) Relationship between bed form wavelength, *L*, and initial mud content for series A–C. (c) Relationship between bed roughness, *k_s_* = 25*H* ^2^/*L*, and initial mud content for series A–C. Error bars represent the variability from the mean across three longitudinal transects. All graphs also show predictions, based on noncohesive sand experiments, as dotted lines (VR1984 and N2014), after *van Rijn* [1984] and *Naqshband et al.* [2014], respectively. The linear fits to *H* and *L* in series A can be used to infer clean sand values of *H* = 83 mm and *L* = 1627 mm.

The experimental results reveal a substantial influence of initial bed mud and EPS content on bed form height (*H*), length (*L*), steepness (*H*/*L*), and bed roughness (*k_s_* = 25*H* ^2^/*L*) [e.g., *van Rijn*, 1984] (Figures 1 and 2 (Figures S3 and S4) and Table 1). When EPS was absent (series A), the final bed topographies show a clear transition from steep, more three‐dimensional, dunes at low mud contents (*m* = 1.9%, A1) to low‐steepness dunes (4.7% < *m* < 11.9%, A2 to A5), and very low steepness dunes (12.7% < *m* < 14.1%, A6 and A7), which are more two‐dimensional, at higher mud contents (Figures 1 and 2). The dunes in runs A4 to A7 contained superimposed current ripples. Bed form height, length, steepness, and roughness all decreased significantly as initial bed mud fraction was increased (Figure 2 (Figure S4) and Table 1). A linear fit to *H* and *L* for series A allows the dimensions of clean sand equivalent bed forms (*m* = 0%) to be estimated at *H* = 83 mm and *L* = 1627 mm.

The addition of EPS to sand‐mud mixtures in series B and C prevented the formation of steep, fully 3‐D, dunes and substantially reduced bed form heights across the same range of initial mud contents as series A. The final bed morphologies for the low EPS cases (approximately 0.03%; Table 1 and Figure 2) showed irregular, low‐steepness, 3‐D dunes. As in series A, bed form height, steepness, and roughness decreased with increasing mud fraction (Figure 2), whereas bed form length remained approximately constant. At low mud fractions, the bed form height, length, steepness, and roughness were smaller than at equivalent mud contents in the EPS‐free experiments (Table 1).

At the higher initial bed EPS fractions examined in series C (0.07–0.1%), the size of the bed forms was reduced substantially (Table 1 and Figures 1 and 2 (Figures S3 and S4)), with bed form types being limited to two‐dimensional ripples for 9.1% < *m* < 12.0% (C1–C3) and a flat bed at *m* = 17.7% (C4). Notably, the dominant bed form height of 4 mm was an order of magnitude smaller than the heights for equivalent mud contents in the absence of EPS (series A). Moreover, in runs C1 to C3, the bed form height, length, steepness, and roughness were independent of *m*.

According to the widely used bed form predictor of *van Rijn* [1984], the dunes in this study should have reached an equilibrium height of 80 mm and an equilibrium length of 2774 mm (Figure 2). With the exception of the bed forms in run A1, which had the lowest mud substratum fraction (*m* = 1.9%), observed dune heights were all lower than the predicted height by up to an order of magnitude. The predicted dune length was also well in excess of all measured lengths, with the difference rapidly increasing as bed mud fraction and bed EPS fraction were increased.

**Figure 3:**
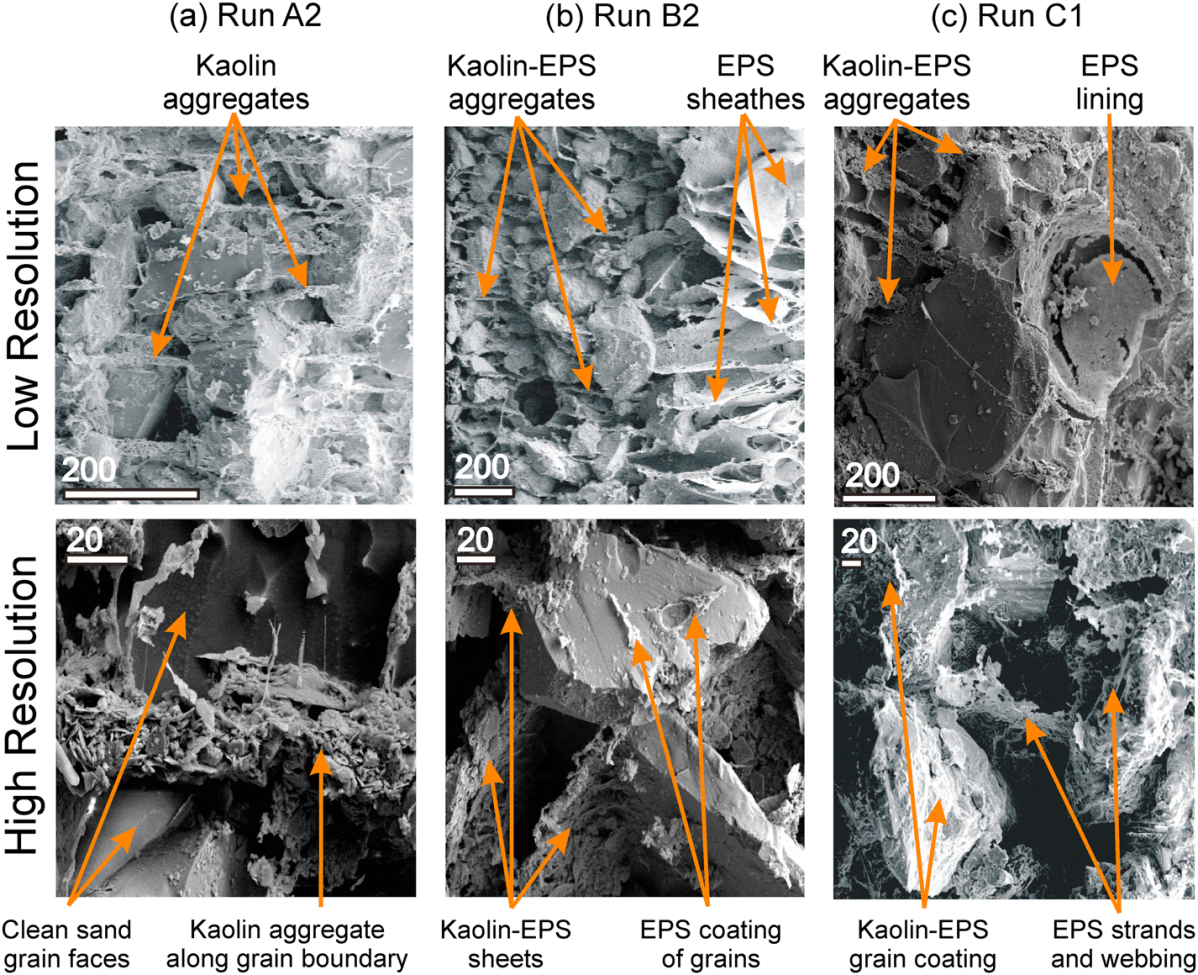
LTSEM images, comparing initial substratum microstructure for selected runs in series A, B, and C. Top and bottom rows show low‐ and high‐resolution images, respectively. Scale bar units are in micrometers. (a) Run A2 (*m* = 4.7%), with plated kaolin particle aggregates found predominantly between sand grains rather than on the exposed sand grain surfaces. (b) Run B2 (*m* = 6.8%; *e* = 0.038%), showing kaolin‐EPS aggregates dominated by EPS sheathes and partial coatings of sand grain surfaces. (c) Run C1 (*m* = 9.1%; *e* = 0.075%), showing EPS lining sand grain socket (top) and EPS strands and webs linking individual sand grains (bottom). Images obtained using procedures outlined in *Tolhurst et al.* [2002].

The initial compositions for selected experimental substrata are compared using LTSEM (Figure 3). In the abiotic samples (run A2; *m* = 4.7%), sand grain surfaces were largely free of clay particles (Figure 3a), and clay platelet aggregates formed distinctive layers between sand grains. At low fractions of EPS (run B1; *m* = 6.8%; *e* = 0.038%), there are EPS‐bound sheaths resembling an “open card structure” (Figure 3b). Aggregates of mud and EPS span voids between grains, and there is a greater adhesion of cohesive material to sand grain surfaces compared with the abiotic (series A) case. At high EPS fractions (run C1; *m* = 9.1%; *e* = 0.075%) the matrix is visibly denser than in series A and B (Figure 3c, top). This density variability is apparent in the void created by the removal of a sand grain that exposed an EPS lining, indicating that the sand grains are enveloped by EPS (in direct contrast to clay‐derived edge‐to‐plate bonds). At higher resolution, strands and webs of EPS link individual sand grains within a matrix (Figure 3c, bottom).

Both the dunes and the ripples migrated during the experiments, resulting in an active layer in the bed, down to a level corresponding to the bed form troughs, and an inactive substratum underneath. Analysis of the postexperiment mud and EPS content in the bed revealed that both components had mostly been removed from the active layer by winnowing, but in the underlying substratum both components remained largely unchanged. This is in agreement with previous experimental work on ripples in mud‐sand and EPS‐sand mixtures [*Baas et al.*, 2013; *Malarkey et al.*, 2015], which indicated that the removal of EPS and mud from the troughs limits bed form growth.

## 4 Discussion

The results presented herein show the dramatic effect that substratum fractions of physically cohesive mud and biologically cohesive EPS have on bed form height, length, and steepness compared with noncohesive sediment substrates that are exposed to similar shear stresses. The addition of mud and EPS significantly reduced both bed form height and length across all the experiments. While substratum mud in isolation has a significant effect, the addition of even small amounts of EPS dominates the combined effect of cohesion and has a dramatic influence on bed form dimensions and bed form type (Figure 2).

### 4.1 Comparison of Physical and Biological Cohesion

The different types of bonding that mud and EPS exert within the substratum can explain the different sensitivities of bed form development to mud‐induced physical cohesion and EPS‐induced biological cohesion. The presence of clay platelet aggregates between the sand grains leads to physical cohesion imparted by mass attractive London‐van der Waals forces and interparticle electrostatic bonding of cohesive particles [*Mehta*, 2014] (Figure 3a). Such electrostatic bonding would increase with salinity, in turn affecting the zeta potential (a measure of the net electrical charge around particles) of clay particles [*Mietta et al.*, 2009]. In contrast, biological cohesion occurs when the polymer creates surface bonding through long chain molecular polymeric strands and gel surface coatings that physically link or envelope the sediment grains (Figures 3b and 3c) [cf. *Underwood and Paterson*, 2003]. In addition, if clay particles and EPS are both present, EPS can enhance the physicochemical cohesive properties of the mud fraction by increasing the molecular attractive forces between clay particles to form physical interparticle bonds that increase the tensile strength of the mud fraction [e.g., *Chenu and Guerif*, 1991]. Although the physicochemical cohesive properties of the mud may be enhanced by EPS, the order of magnitude changes in bed form properties suggests that the polymeric strands and gel surface coatings are the dominant mechanism for enhanced cohesion. This enhanced cohesion restricts the heights and lengths of mixed mud‐EPS bed forms, and high levels of EPS modify the bed form type from dunes to ripples and ultimately flat beds. Flow separation in the lee of bed forms is important for their development, in particular at the point where the shear layer between the main flow and the vortex reattaches to the bed, controlling substratum erosion. It is postulated that the cohesive strength of the bed limits the ability of the flow to erode sediment, thus limiting the height to which the bed form can ultimately grow.

### 4.2 Implications for Modern Environments

The changes in dominant bed form geometries and dimensions described herein have a number of important implications for morphodynamics, sediment transport, and biophysical habitat modeling. Our results demonstrate that form roughness (*k_s_* = 25*H* ^2^/*L*) in numerical models may be overestimated by as much as 2 orders of magnitude in areas of significant biophysical cohesion. Models that overpredict bed roughness would underestimate flow velocities and overestimate background turbulence levels, which in turn has implications for flocculation processes, sediment fluxes, and ultimately morphodynamic change in regional models [e.g., *Sutherland et al.*, 2004]. Our experimental data also suggest that bed form scour depths may be overestimated, by up to an order of magnitude, in engineering design of bridge piers, pylons, buried pipelines, and cables, because bed forms with natural biophysical cohesion do not reach the heights and trough scour depths as those produced in cohesionless sand. Similarly, maintenance of navigable channels by dredging requires information on the highest crest elevations for operational under keel clearance [*van der Mark et al.*, 2008], which may be overpredicted by up to an order of magnitude.

The experimental data presented herein also suggest that sediment transport rates may vary significantly in sediment beds containing EPS and at scales that could be relevant for the dynamics of biological activity [e.g., *Maddock*, 1999]. The need to couple biological habitat models with simulations of time‐varying hydrodynamics and morphodynamics has recently been highlighted [*Hauer et al.*, 2009]. The results presented herein indicate that biological hydrodynamic coupling requires an understanding of how bed form dimensions influence habitat availability and the distribution of sediments and organisms. Furthermore, the results also indicate that microbial communities could play a key role in reducing bed form dimensions through the secretion of EPS, a system feedback that is not presently incorporated in morphodynamic and biological predictions.

### 4.3 Implications for Ancient Environments

The effects of cohesion on bed form size and shape have significant implications for paleohydraulic and paleoenvironmental interpretations. Ancient strata record the modification of sedimentary environments, and the formation of characteristic sedimentary structures, by microfauna and meiofauna since the first appearance of microbial life in the Precambrian [*Schopf*, 1992], and the expansion and diversification of microbial life to specific environments, such as tidal flats, in the Phanerozoic [*Ericksson et al.*, 2004]. This geological evidence is typically based on sedimentary facies where microfauna are present in large quantities, such as in microbial mats and stromatolites. However, smaller communities of microbial life may have influenced sedimentary processes in a wider range of settings during the Precambrian and possibly also earlier in the Precambrian than is detectable by direct fossil evidence. The results of the present experiments suggest that dune dimensions and thus their cross‐set thicknesses are reduced even at low mud and extremely low EPS fractions. This observation may provide a tool for framing the search for early life on Earth to indirect evidence from the shape and average size of subaqueous dunes in the early Precambrian. This approach would rely on an improved quantitative understanding of how bed form cross‐set thickness in mixed sand‐mud is related to the height of bed forms, as such understanding in clean sand underpins prediction of bed form height from the thickness distribution of ancient cross sets [e.g., *Paola and Borgman*, 1991; *Leclair and Bridge*, 2001]. The experimental data presented herein suggest that the scales of preserved bed form sets and cross stratification within deposits formed in natural substrates containing sand, mud, and microbiota are likely to diverge significantly from those established for substrates devoid of such cohesion. Moreover, if not accounted for, the reduction in bed form size and steepness in response to physical and biological cohesion may lead to flaws in the reconstruction of paleohydraulic variables, such as flow discharge and channel depth and width [e.g., *Rubin and Carter*, 2006], which has already been suggested elsewhere for biofilms formed by cyanobacteria [*Hagadorn and McDowell*, 2012]. For instance, ancient ripple cosets could be misinterpreted as being produced by low flow velocities, based on cohesionsionless predictions, when high levels of substrate cohesion would suggest much higher flow velocities.

## 5 Conclusions

Our experiments examined the importance of combined physical and biological cohesion on current‐generated bed form morphology. The experimental data reveal that both have significant influence and that biologically produced extracellular polymeric substances (EPS) are by far the most effective of the two components in reducing bed form dimensions and steepness, due to their stronger interparticle bonding. The combined effect of biological and physical cohesion has been shown to alter bed form dimensions by up to an order of magnitude and bed roughness by up to 2 orders of magnitude. These large changes result from the suppression of dunes in favor of ripples as the dominant bed form. Changes induced by physical and biological cohesion render existing and widely adopted bed form predictors, based on cohesionless grains, inadequate for many naturally occurring sedimentary environments, particularly coastal and estuarine systems that tend to comprise significant levels of biologically active fine‐grained sediment.

The present results have significant implications for modern coastal management and engineering, the interpretation of ancient sedimentary environments, and the role of biological mediation in such sedimentary systems. Physical and biological cohesion require incorporation into new generations of bed form predictors, morphodynamic models, and biological habitat models. The results also provide a basis for reassessing the impact of early life on bed form dynamics and sedimentary systems more generally.

## Supporting information

Supporting Information S1
