# Supplementary material for: The role of biophysical cohesion on subaqueous bed form size
Source: Geophys Res Lett. 2016 Feb 19;43(4):1566–73. doi: 10.1002/2016GL067667 (PMC4794777; doi:10.1002/2016GL067667)
Supplement: Supplementary file 1 — Supporting Information S1 [file GRL-43-1566-s001.pdf]

AGU PUBLICATIONS

Geophysical Research Letters

Supporting Information for

The role of biophysical cohesion on subaqueous bed form size

Daniel R. Parsons[1], Robert J. Schindler[1,2], Julie A. Hope[3], Jonathan Malarkey[4], Jaco H. Baas[4], Jeffrey Peakall[5], Andrew J. Manning[1,2,6], Leiping Ye[1], Steve Simmons[1], David M. Paterson[3], Rebecca J. Aspden[3], Sarah J. Bass[2], Alan G. Davies[7], Ian D. Lichtman[4,8], Peter D. Thorne[8]

[1]Department of Geography, Environment and Earth Sciences, University of Hull, Hull, UK, [2]School of Marine Science and Engineering, Plymouth University, Plymouth, UK, [3]School of Biology, University of St. Andrews, Saint Andrews, UK, [4]School of Ocean Sciences, Bangor University, Anglesey, UK, [5]School of Earth and Environment, University of Leeds, Leeds, UK, [6]HR Wallingford, Wallingford, UK, [7]Centre for Applied Marine Sciences, Bangor University, Anglesey, UK, [8]National Oceanography Centre, Liverpool, UK

Contents of this file

Figures S1 to S4

Introduction

There are four additional figures that provide supporting information on the set up of the experiments and additional results from the experiments including additional contour maps from the full suite of runs and photographs of the final bed form states.

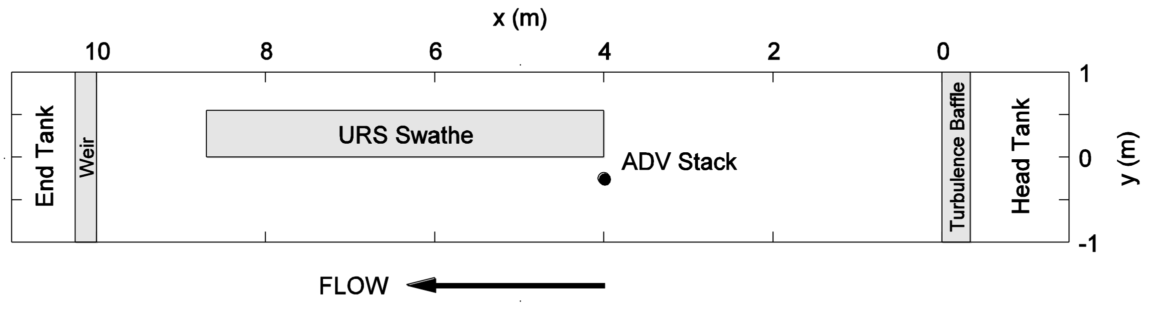

Figure S1. Plan view of the recirculating flume. Flow rises in the head tank and passes through a turbulence baffle before entering the channel. A weir at the distal end maintains water surface slope. Dune morphology was measured along a 4.7 m central swathe 4.0 ≤ x ≤ 8.7, 0 ≤ y ≤ 0.55 m and flow was monitored using four stacked ADVs close to the centerline at x = 4.0 m.

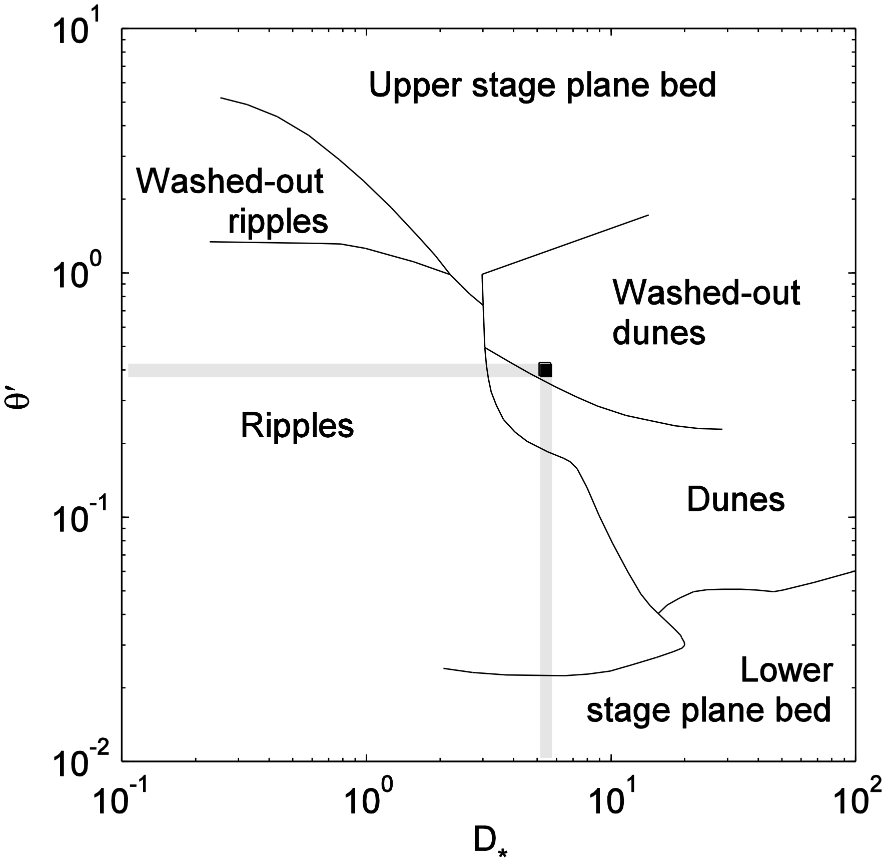

Figure S2. Bed form phase diagram, showing the relationship between sediment characteristics, flow forcing and equilibrium bed form type across the ripple-dune transition. The mobility parameter $\theta' = \rho U^2/[(s-1)C'^2 D_{50}]$ (U is the flow velocity, s is the ratio of sediment density to water density, C' is the Chézy coefficient related to grain roughness, and D_{50} is the median grain diameter) and the non-dimensional particle diameter $D_* = D_{50}[g(s-1)/v^2]^{1/3}$ (g is the acceleration due to gravity, and v is the kinematic viscosity) vary with the grain size distribution of each substrate. θ' is 0.38 for the sand fraction only, and increases to 0.43 for m = 17.7%. At the mean water temperature of 17.5 °C, D_* is 5.7 for the sand fraction only, and D_* is 5.1 for m = 17.7%. Equivalent values for D_{50} are 0.239 mm and 0.214 mm, respectively. Despite these variations, dunes are predicted in all cases, indicated by the experimental phase space. Modified after *van den Berg and van Gelder* [1993].

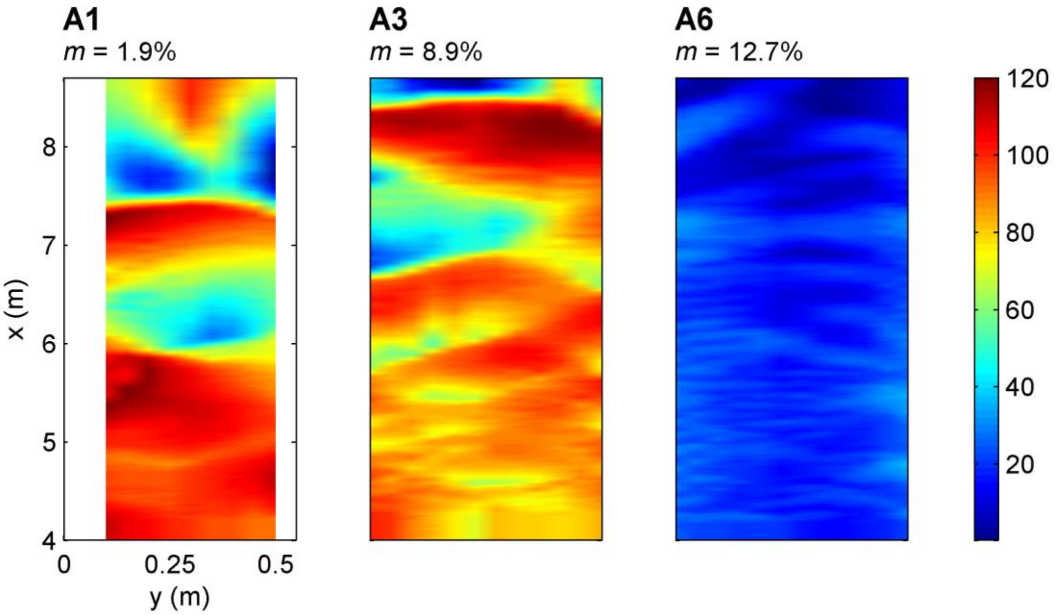

Figure S3. Additional planform contour maps of the final bed morphology of the experimental runs from series A (no EPS) not shown in Figure 1, where a reduction in bed form dimensions occurs as mud content is increased, resulting in a transition from fully three-dimensional dune-scale bed forms via current ripples to surfaces that approach a flat bed across the series of runs. *m* = initial bed mud content.

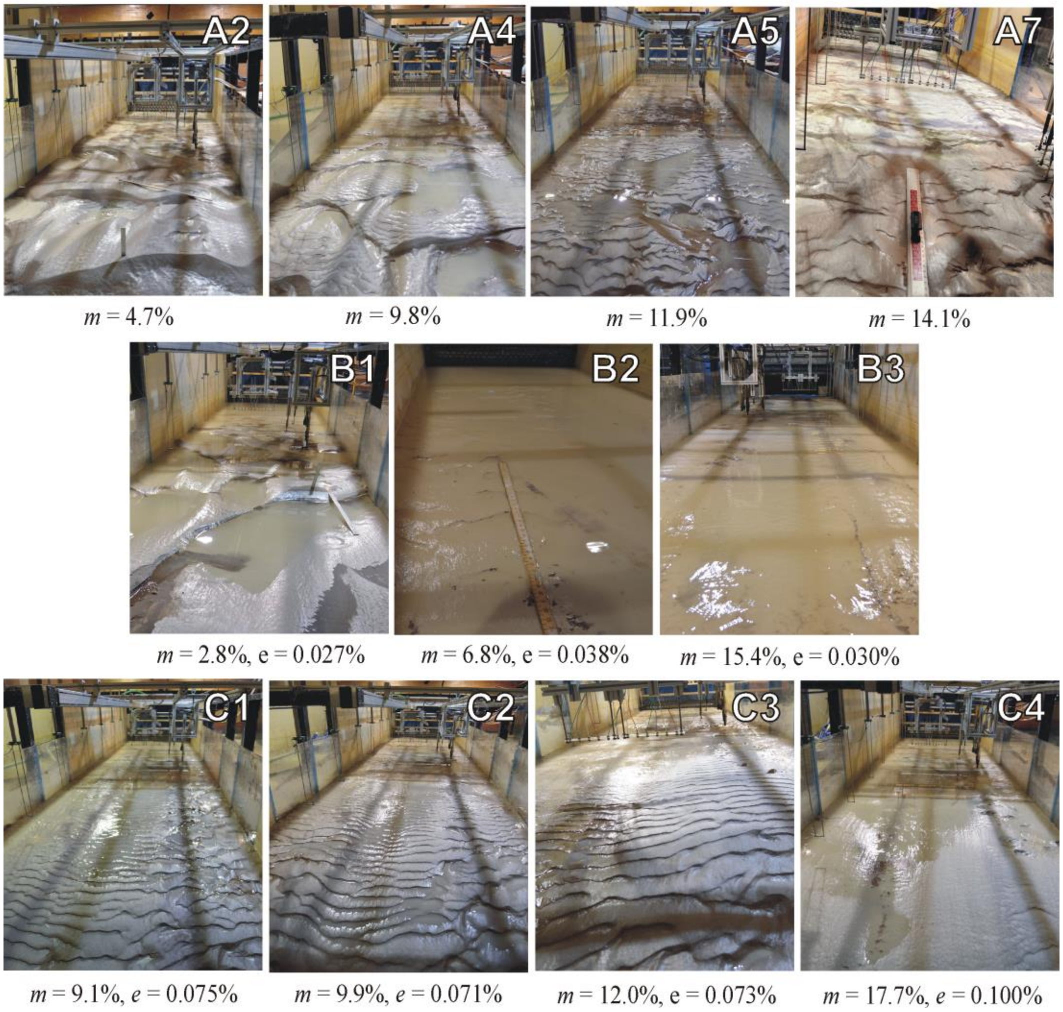

Figure S4. Images of final bed morphology of the experimental runs taken over the measurement domain from the distal end of the flume. First row shows selected runs for series A (no EPS), where a reduction in bed form dimensions occurs as mud content is increased, resulting in a clear transition from fully three-dimensional dune-scale bed forms via current ripples to surfaces that approach a flat bed. The second row shows bed forms from series B (low EPS). These bed forms are small compared with series A, and a transition from irregular, low-steepness 3D dunes (run B1) to an almost featureless surface (run B3) is evident. The third row show bed forms from series C (high EPS). These bed forms are limited to 2D ripples and approach a featureless surface at the highest mud content (run C4). *m* = initial bed mud content, *e* = initial bed EPS content. Note the dramatic changes in bed form type and size for mere trace amounts of EPS. All panels show flow towards the observer.
